# Survival after traumatic cardiac arrest is possible—a comparison of German patient-registries

**DOI:** 10.1186/s12873-022-00714-5

**Published:** 2022-09-10

**Authors:** Stephan Seewald, Jan Wnent, Jan-Thorsten Gräsner, Ingvild Tjelmeland, Matthias Fischer, Andreas Bohn, Bertil Bouillon, Holger Maurer, Rolf Lefering

**Affiliations:** 1 Institute for Emergency Medicine, University Hospital Schleswig-Holstein, Arnold-Heller-Straße 3, buildung 808, 24105 Kiel, Germany; 2 Department of Anesthesiology and Intensive Care Medicine, University Hospital Schleswig-Holstein, Kiel, Germany; 3 School of Medicine, University of Namibia, Windhoek, Namibia; 4 Faculty of Medicine, Institute of Clinical Medicine, University of Oslo, Oslo, Norway; 5 Division of Prehospital Services, Oslo University Hospital, Oslo, Norway; 6 Department of Anesthesiology, Intensive Care Medicine and Emergency Medicine, ALB-FILS Kliniken, Goeppingen, Germany; 7 City of Muenster, Fire Department, Muenster, Germany; 8 Department of Anesthesiology, Intensive Care and Pain Medicine, University Hospital Munster, Munster, Germany; 9 Department of Trauma and Orthopedic Surgery, Cologne Merheim Medical Center, Cologne, Germany; 10 Department of Anesthesiology and Intensive Care Medicine, University Hospital Schleswig-Holstein, Luebeck, Germany; 11 Institute for Research in Operative Medicine, Faculty of Health, University of Witten/Herdecke, Witten, Germany

**Keywords:** Traumatic cardiac arrest, Resuscitation, German resuscitation registry, GRR, Trauma registry, TR-DGU

## Abstract

**Background:**

Out-of-hospital cardiac arrest (OHCA) due to trauma is rare, and survival in this group is infrequent. Over the last decades, several new procedures have been implemented to increase survival, and a “Special circumstances chapter” was included in the European Resuscitation Council (ERC) guidelines in 2015. This article analysed outcomes after traumatic cardiac arrest in Germany using data from the German Resuscitation Registry (GRR) and the TraumaRegister DGU® (TR-DGU) of the German Trauma Society.

**Methods:**

In this study, data from patients with OHCA between 01.01.2014 and 31.12.2019 secondary to major trauma and where cardiopulmonary resuscitation (CPR) was started were eligible for inclusion. Endpoints were return of spontaneous circulation (ROSC), hospital admission with ROSC and survival to hospital discharge.

**Results:**

1.049 patients were eligible for inclusion. ROSC was achieved in 28.7% of the patients, 240 patients (22.9%) were admitted to hospital with ROSC and 147 (14.0%) with ongoing CPR. 643 (67.8%) patients were declared dead on scene. Of all patients resuscitated after traumatic OHCA, 27.3% (259) died in hospital. The overall mortality was 95.0% and 5.0% survived to hospital discharge (47). In a multivariate logistic regression analysis; age, sex, injury severity score (ISS), head injury, found in cardiac arrest, shock on admission, blood transfusion, CPR in emergency room (ER), emergency surgery and initial electrocardiogram (ECG), were independent predictors of mortality.

**Conclusion:**

Traumatic cardiac arrest was an infrequent event with low overall survival. The mortality has remained unchanged over the last decades in Germany. Additional efforts are necessary to identify reversible cardiac arrest causes and provide targeted trauma resuscitation on scene.

**Trial registration:**

DRKS, DRKS-ID DRKS00027944. Retrospectively registered 03/02/2022.

**Supplementary Information:**

The online version contains supplementary material available at 10.1186/s12873-022-00714-5.

## Background

Out-of-hospital cardiac arrest (OHCA) has an incidence of 36 to 244/100,000 inhabitants/year in Europe [1]. It is commonly treated by emergency medical services (EMS) either in a paramedic or a physician-based system. The majority of OHCA occur because of a cardiac event, described as a cardiac cause [1]. Traumatic cause of cardiac arrest (CA) is a rare entity in Germany and other European countries. According to the annual report from the German Resuscitation Registry (GRR), only 3% of the reported cardiac arrests were due to major trauma [2]. In the European Registry of Cardiac Arrest (EuReCa)-ONE study, Gräsner et al. reported 4.1% of the cases with major trauma as cause of arrest. Survival with good neurological outcome after traumatic cardiac arrest remained low, and patients were younger than patients with cardiac arrest of a cardiac origin [3–6].

In 2015 the European Resuscitation Council (ERC) published a special section in the guidelines focusing on resuscitation in traumatised patients, emphasising treatment of potentially reversible causes of cardiac arrest (e.g. hypovolemia, tension pneumothorax, pericardial tamponade, and hypoxemia) [7]. In the current 2020/2021 resuscitation guidelines, several new or re-invented techniques were recommended as standard practice in trauma resuscitation [8]. In cases with severe bleeding, trauma tourniquets, pelvic slings and hemostyptics were widely-used [9]. In some regions, more invasive techniques like “Resuscitative endovascular balloon occlusion of the aorta “(REBOA) or “Clamshell-thoracotomy” has been implemented [10, 11].

The American College of Surgeons (ACSCOT) and the National Association of EMS Physicians (NAEMSP) recommended withholding resuscitation in situations where death is inevitable or in trauma patients presenting with apnea, pulselessness and without organised electrocardiogram (ECG) activity [12]. The current ERC guidelines were more restrictive and recommended that termination of CPR should be considered if there is no return of spontaneous circulation (ROSC) after reversible causes have been addressed or no detectable ultrasonographic cardiac activity in pulseless electrical activity (PEA) after reversible causes have been addressed [8].

After a first study from the German Resuscitation Registry and the TraumaRegister DGU® was published in 2011, this study investigated whether the outcome (ROSC, ROSC at hospital admission, survival to hospital discharge) after a traumatic cardiac arrest had improved. Beneficial effect on patient survival was expected as specific resuscitation algorithms have been widely applied nowadays.

## Methods

This study is a joint project of the TraumaRegister DGU® (TR-DGU) and the German Resuscitation Registry (GRR).

### German resuscitation registry

The German Resuscitation Registry (GRR) is a voluntary registry founded in 2002 and is run by the German Society of Anesthesiology and Intensive Care Medicine (Deutsche Gesellschaft für Anästhesiologie und Intensivmedizin e.V., DGAI). Each EMS or hospital can decide whether they want to participate in GRR. There is no legal obligation to participate*.* The GRR collects anonymous data on out-of-hospital and in-hospital cardiac arrest patients. The data is collected at different time points “pre-hospital treatment/initial treatment”, “in-hospital treatment”, and “long-term survival”. The datasets adhere to the Utstein recommendations [13, 14], and data are entered into the database via a password-protected online reporting system. The database includes plausibility and completeness checks as well as an analysing tool. Participating EMS and hospitals receive a comprehensive report, including risk-stratification with the ROSC-after-cardiac-arrest-score (RACA) or the CaRdiac-Arrest-Survival-Score (CRASS) [15, 16]. GRR receives information from EMS systems covering approximately 30 million inhabitants, 36% of Germany’s total population of 83 million. This project is approved by the scientific advisory board of GRR (Ref. No.: 20190601_JW).

### TraumaRegister DGU®

The TraumaRegister DGU® (TR-DGU) of the German Trauma Society (Deutsche Gesellschaft für Unfallchirurgie e.V., DGU) was founded in 1993. This multi-centre database provides pseudonymised information about severely injured patients. Data are collected prospectively from the site of the accident until discharge from hospital at the following times: A) pre-hospital phase, B) emergency room and initial surgery, C) intensive care unit and D) discharge. The documentation includes detailed information on demographics, injury patterns, comorbidities, pre- and in-hospital management, treatment and care in the intensive care unit (ICU), relevant laboratory findings and data on transfusion and outcome of each individual. Inclusion criteria in the registry are admission to hospital via emergency room with subsequent care in an ICU or admitted to hospital with vital signs but dead before admission to ICU.

Scientific data analysis of this project is approved according to a peer-review process and it is in line with the publication guidelines of the TR-DGU and registered as TR-DGU project ID 2018–043.

### Patients

Patients suffering OHCA due to trauma between 01.01.2014 and 31.12.2019, where cardiopulmonary resuscitation (CPR) was started, and the data was registered either in the GRR or in TR-DGU were eligible for inclusion. The data from both registries were not matched or merged; analyses were performed independently and in parallel. Due to data security and confidentiality, only anonymised data was available in both registries, and there was no information available about whether or not a patient was included in both registries. GRR provided information about the out-of-hospital treatment and outcome after cardiac arrest, but the TR-DGU was limited to trauma patients arriving in the hospital. The dataset in GRR focused on resuscitation related items, and the dataset in TR-DGU focused on surgery-related items. In this analysis, we used data from these two large national registries to analyse the midterm outcome of CPR after traumatic cardiac arrest in Germany.

To ensure high-quality data, we only included EMS systems from GRR with:

- incidence of resuscitation started > 30/100,000 inhabitants per year,
- any return of spontaneous circulation (ROSC) < 80%,
- ROSC-After-Cardiac-Arrest-score (RACA-score) available for > 60% of the patients [15],
- documentation of in-hospital care (in case of hospital admission) for > 30% of the patients.

First, all included cases in GRR were analysed. Primary endpoints were ROSC and hospital admission with ROSC. The secondary endpoints were hospital mortality and discharge with a good neurological outcome (Cerebral Performance Categories (CPC) 1 or 2).

Second, all primary admissions from TR-DGU treated in participating German hospitals with information about outcomes were included (*n* = 178,141). A total of 4,147 (2.7%) patients had a cardiac arrest and cardiopulmonary resuscitation on scene. Patients with missing data for blood pressure (BP) or heart rate (HR) on hospital admission (*n* = 637) and those with ongoing CPR (*n* = 842) were excluded. Patients with missing trauma mechanism (*n* = 46) and those with a mechanism other than trauma (*n* = 180) were also excluded, leaving 2,460 cases with OHCA after trauma for analysis (1.4%).

In summary, the included data from GRR consisted of all OHCA cases attributed to trauma in the high data quality group (irrespective of outcome). The TR-DGU data consisted of patients with successful prehospital resuscitation who arrived at hospital with ROSC.

### Statistical analysis

Continuous data were presented as mean with standard deviation (SD) or median with quartiles in case of skewed distribution. Statistical comparisons were performed with Mann–Whitney U-test. Categorical data were presented as number of patients with percentages (%), and differences were evaluated with chi-squared test. *P*-values < 0.05 were considered statistically significant.

We developed two multivariate logistic regression models to identify risk factors for in-hospital mortality (dependent variable). The model was developed mainly on clinical significance of the predictor variables. Experts and statisticians from both registries (CPR and trauma) were involved. The model in the TR-DGU dataset considered classical predictors from trauma research (age, sex, penetrating mechanism, unconsciousness, shock, Injury Severity Score, injured body regions: head, thorax, abdomen, extremities), known risk factors from previous research in traumatic CA [3] (patient found in CA; repeated CA in the emergency room), early interventions in the hospital (blood transfusion, emergency surgery) and hospital level of care. Non-significant predictors (*p* > 0.05) were deleted from the final model.

The model in the GRR dataset considered classical predictors from CA research [15] (age, sex, bystander CPR, initial ECG, found in CA, location of CA) with relevant impact in univariate analysis (*p* < 0.5) and status at hospital admission (shock at admission).

In GRR, data were first used to analyse all patients with CPR started and then the subgroup of patients with ROSC on admission only, similar to the TR-DGU approach. TR-DGU data were analysed for patients with ROSC on hospital admission only. Results were presented as odds ratios (OR) with 95% confidence intervals. Analyses were performed using SPSS statistical software (version 26, IBM Inc., Armonk, NY, USA).

The ethics committee at Christian-Albrechts University in Kiel provided ethical approval for this study (Ref. No.: D 497/2019).

## Results

**Fig. 1:**
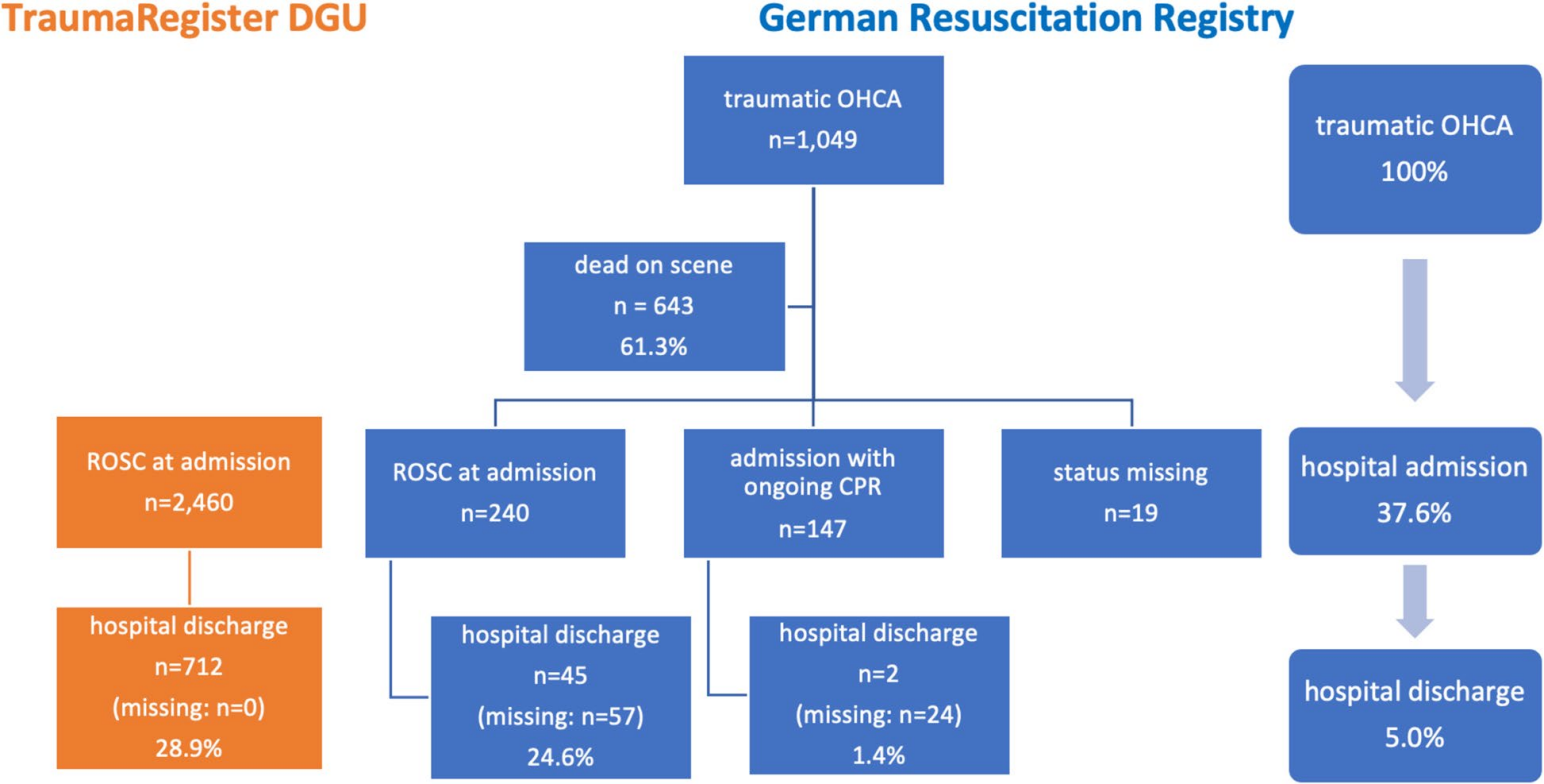
Flow chart of survival after traumatic cardiac arrest based on GRR and TR-DGU. GRR, German Resuscitation Registry; OHCA, out-of-hospital cardiac arrest; ROSC, return of spontaneous circulation; TR-DGU, Trauma Registry of the German Society for Trauma Surgery

In the study period, 60,545 patients suffered an OHCA and 2,419 (4.0%) had trauma reported as the cause of arrest. In 1,049 (43.4%) of those patients, CPR was started, and these patients were included in this analysis (Fig. 1). The majority of patients were male (75.1%), 55.0% of the events occurred in the street, 42.2% were witnessed by a bystander, and bystanders started CPR in 34.9% of the cases. Primary heart rhythm was asystole in 65.9%, PEA in 29.1% and ventricular fibrillation (VF) in 5.0% of the cases. The mean time from call to EMS arrival was 8.8 min (SD 5.2 min) and the mean time on scene was 37.8 min (SD 14.9 min) (Table 1).

**Table 1:** Characteristics of the included cases from both registries

Source	All patients with traumatic CA and CPR started	Admitted to hospital with ROSC	Admitted to hospital with ROSC
	GRR group of high-quality data	GRR group of high-quality data	TR-DGU
No. of cases	*n* = 1,049	*n* = 240	*n* = 2,460
Time period	2014–2019 6 years	2014–2019 6 years	2014–19 6 years
Age in years: median [quartiles]	55 [35–73]	58 [35–76]	57 [39–74]
Male sex	75.1%	70.4%	73.0%
Mechanism			
Traffic – car/lorry			18.6%
Traffic—motorbike			10.4%
Traffic – bicycle			9.4%
Traffic – pedestrian			8.4%
Traffic—other			2.3%
High fall			14.9%
Low fall (< 3 m)			25.8%
Scene of cardiac arrest			
Home	20.8%	31.8%	
Nursing home	2.0%	1.7%	
Workplace	5.0%	7.9%	
Street	55.0%	42.7%	46.8% (traffic)
Public place	9.8%	9.2%	
Medical institution^b^	2.2%	2.5%	
Public event	0.2%	0.4%	
Other	5.0%	3.8%	
Missing data (n)	3	1	
ECG			
VF	5.0%	10.6%	
PEA	29.1%	44.7%	
Asystole	65.9%	44.7%	
missing data (n)	11	5	
CA witnessed			
By laypeople	42.2%	45.4%	
By EMS	12.2%	14.6%	
Not witnessed	45.6%	40.0%	
Bystander CPR (if not witnessed by EMS)	34.9%	32.2%	
Use of defibrillator	12.2%	17.5%	
ROSC	28.7%	100%	
Status on admission			
ROSC	240 (22.9%)	100%	100%
Ongoing CPR	147 (14.0%)		
Died on scene	643 (61.3%)		
Missing	19 (1.8%)		
Pre-hospital intubation	682 (65.0%)	82.5%	90.6%
Transportation by helicopter			27.2%
Time from call to EMS arrival in min: mean (SD)	8.8 (5.2)	8.8 (4.4)	
Time from call to EMS arrival in min – missing	*n* = 33	*n* = 5	
Time on scene in min (when transport to hospital has been initiated): mean (SD)	37.8 (14.9)	40.0 (15.0)	35.3 (18.5)^a^
Time on scene – missing	*n* = 56	*n* = 19	*n* = 563^a^
Time from accident to hospital admission in min: mean (SD)	60.1 (21.8)	63.6 (22.9)	69.7 (29.1)
Time from accident to hospital – missing	*n* = 54	*n* = 20	*n* = 301
Injury Severity Score: mean (SD)			35.6 (20.2)
Injury Severity Score: median [quartiles]			33 [21–50]
Again CA/CPR in ER			28.2%^a^
Time in the ER in min: mean (SD)			68.7 (56.5)^a^
Admitted to ICU			81.6%
Died in the ER			16.4%
Declared dead on scene	643 (67.8%)	0	
Died in hospital	259 (27.3%)	138 (75.4%)	71.1%
Died overall	902 (95.0%)		
Died – missing data (n)	100	57	0
CPC 1 or 2 at discharge (survivor only)	25 of 32 (78.1%)	25 of 32 (78.1%)	383 of 696 (55.0%) (adapted GOS 4 + 5)
CPC – missing data	15	13	16

*CA* Cardiac arrest, *CPC* Cerebral performance category, *CPR* Cardiopulmonary resuscitation, *EMS* Emergency medical service, *ER* Emergency room, *GOS* Glasgow outcome scale, *GRR* German Resuscitation Registry, *PEA* Pulseless electrical activity, *SD* Standard deviation, *ICU* Intensive care unit, *ROSC* Return of spontaneous circulation, *VF* Ventricular fibrillation

^a^available only for TR-DGU patients with standard documentation (56%)

^b^includes doctors’ offices and smaller rehabilitation clinics and affiliated hospitals that do not provide their own resuscitation team but alert the EMS in such a case

Any ROSC was achieved in 301 (28.7%) patients. In terms of status on hospital admission, 240 patients (22.9%) were admitted with ROSC and 147 (14.0%) with ongoing CPR. In the majority of cases, 67.8% (643) were declared dead on scene. Of all patients with traumatic OHCA where CPR was attempted, 27.3% (259) died in hospital. The overall mortality was 95.0% (*n* = 902).

Out of the 240 patients admitted to hospital with ROSC in GRR, 82.5% were intubated on scene, 138 patients (75.4%) died in the hospital, and 45 patients survived to hospital discharge (missing data: *n* = 57). Data on neurological status at hospital discharge was available for 31 patients (missing data: *n* = 14); 25 survived with CPC 1 or 2. In the GRR group, survivors had a significantly higher rate of PEA or VF as first rhythm, cardiac arrest was witnessed either by a layperson or EMS, occurred more often at home, and the time from CPR started to first ROSC was significantly shorter. There was no difference in sex, age, bystander CPR rate, time on scene and time to hospital admission (Table S1). If only patients with ROSC on admission were included in the analysis, no difference was found between survivors and non-survivors based on the GRR group (Table S2).

In TR-DGU, survivors were younger, more often male, less frequently found in cardiac arrest, had lower Injury Severity Score (ISS), were less often admitted in shock, received less frequently blood transfusion, and had a lower rate of recurrent cardiac arrest during treatment in the emergency department compared to the patients in GRR (Table 2).

**Table 2:** Survivor versus non-survivor in patients admitted to hospital with ROSC (*n* = 2460; source: TR-DGU)

	Survivor	Non-survivor	*p*-value
	*n* = 712	*n* = 1,748	
Age (years)^a^	55 [37–69]	59 [40–76]	< .001
Male sex	78.7%	70.7%	< .001
Pre-injury ASA 3–4	24.9%	27.3%	.238
Traffic	46.8%	46.9%	.92
High fall	14.9%	14.6%	.88
Low fall (< 3 m)	23.0%	25.8%	.15
Found in CA	31.1%	48.8%	< .001
Injury Severity Score^b^	27.5 (18.0)	38.9 (20.3)	< .001
Head injury (AIS 3 +)	56.5%	62.8%	< .001
Thorax injury (AIS 3 +)	53.4%	59.8%	.004
Abdominal injury (AIS 3 +)	10.0%	14.9%	.001
Extremity injury (AIS 3 +)	21.5%	22.2%	.70
Penetrating mechanism	3.5%	4.7%	.17
Shock (sBP < = 90) on admission	24.9%	45.3%	< .001
CA during ER treatment	7.9%	36.6%	< .001
Blood transfusion	16.3%	29.7%	< .001
Emergency surgery	28.8%	25.4%	.083
Admitted to ICU	98.5%	74.7%	< .001
LOS in hospital (days)^a^	21 [12–34]	1 [1–4]	< .001

*AIS* Abbreviated Injury Scale, *ASA* American Society of Anesthesiologists Physical Status Classification System, *CA* Cardiac arrest, *ER* Emergency room, *ICU* Intensive care unit, *LOS* Length of stay, *sBP* Systolic blood pressure, *SD* Standard deviation, *TR-DGU* Trauma Registry of the German Society for Trauma Surgery

Continuous measurements are presented as ^a^median [quartiles] or ^b^mean (SD)

In the multivariate logistic regression model, hospital mortality was the dependent variable and age group, sex, American Society of Anesthesiologists (ASA) category 3 and 4, initial OHCA, cardiac arrest/CPR in the emergency room (ER), head injury, thoracic injury, abdominal injury, ISS, blood transfusion, fall from a low height and emergency surgery, were independent variables. The ASA category, thoracic injury, abdominal injury, and fall from low height were excluded during model building. The final model is shown in Table 3 and reached Nagelkerke’s R^2^ of 0.273. Cardiac arrest in ER (OR 5.70 (95% CI 3.78–8.70)) and age above 80 years (OR 4.22 (95% CI 2.97–6.00)) were identified to be high-risk factors for in-hospital mortality, while head injury, initial OHCA, blood transfusion, shock on admission, and age between 70 and 79 years were classified as relevant risk factors (OR 1.46 (95% CI 1.10–1.93)).

**Table 3:** Final model of the multivariate logistic regression analysis to predict mortality in patients admitted to hospital with ROSC (*n* = 2,460; source: TR-DGU)

Variable	Unit	Coefficient	*p*-value	OR	95% CI
Age (years) (reference 0-59)	60–69	+ 0.22	.126	1.25	0.94–1.66
	70–79	+ 0.38	.009	1.46	1.10–1.93
	80 +	+ 1.44	< .001	4.22	2.97–6.00
Sex	male	- 0.28	.023	0.76	0.60–0.96
ISS	per point	+ 0.025	< .001	1.025	1.018–1.032
Head injury	AIS 3 +	+ 0.78	< .001	2.17	1.71–2.76
Extremity injury	AIS 3 +	- 0.46	0.001	0.63	0.48–0.84
Found in CA	yes	+ 0.74	< .001	2.10	1.70–2.59
Shock on admission	yes	+ 0.65	< .001	1.91	1.53–2.39
Blood transfusion	yes	+ 0.71	< .001	2.03	1.51–2.72
CA/CPR in the ER (reference: no)	yes	+ 1.74	< .001	5.70	3.74–8.70
	unknown	+ 0,40	0.001	1.50	1.19–1.86
Emergency surgery	yes	- 0.31	.018	0.74	0.57–0.95
Level 1 hospital	yes	- 0.27	0.041	0.77	0.60–0.99
Constant		-1.22	< .001		

*CA* Cardiac arrest, *CPR* Cardiopulmonary resuscitation, *ER* Emergency room, *Level 1 hospital* supra-regional (certified) trauma center with at least 50 ISS 16 + cases per year, all trauma centers in Germany are classified as Level 1, 2 (regional) or 3 (local) based on a structured auditing which is updated every 3 years, *ISS* Injury severity score, *TR-DGU* Trauma Registry of the German Society for Trauma Surgery

The same multivariate logistic regression analysis was performed in the GRR group admitted with ROSC on admission to hospital. Included as independent variables were age group, sex, bystander CPR, location of arrest, witnessed cardiac arrest, shock on admission and initial electrocardiogram (ECG) rhythm. Only the initial ECG rhythm could be found as a predictor of mortality. The Nagelkerke’s R^2^ was 0.107 (table S3).

## Discussion

OHCA caused by trauma was rare compared to a presumed cardiac cause, and survival was significantly lower [5]. In the present study, only 4.0% of the patients in GRR suffered an OHCA secondary to trauma. In comparison, cardiac arrest with presumed cardiac cause was 60.6% in 2019 [2]. In TR-DGU, only 2.3% of the cases had a cardiac arrest before EMS arrival or during EMS treatment. This proportion has decreased compared to a historical control group from 1993 to 2004, where 415 out of 10,359 patients (4.0%) [17] had presumed cause of cardiac arrest registered as trauma. Survival in our study (5.0%) was similar to results reported from previous studies in Germany [3]. However, outcome information from approximately 10% of patients was missing in this study, leaving some uncertainty about true mortality.

The survival rate reported here was high compared with data from France published by Escutnaire and colleagues [5]. They found that 1.5% of the patients in the trauma group survived and 5.9% in the medical group [5]. The Trauma Audit and Research Network (TARN) database reported an in-hospital 30 days mortality of 92.5% between 2009 and 2015. Included in this study were patients with admission to hospital for three days or longer, intensive or high dependency care or transfer for further specialist care [4]. The reported patients sustained less severe injuries (median ISS 29) and were about ten years younger than our study population.

A direct comparison between GRR and TR-DGU was difficult due to the different starting points of data collection and the different inclusion and exclusion criteria of both registries. However, by comparing data derived from both registries, we could overview the extent to which survival and survival with favourable neurological outcomes after OHCA caused by trauma was possible and which factors influenced the outcome. Interestingly, overall survival for those admitted to hospital with ROSC was comparable in both registries. Regardless of the changes in resuscitation guidelines towards a more specialised treatment of traumatic cardiac arrest in 2015 [7], survival had not significantly improved in Germany. One possible explanation could be that guideline implementation usually takes a long time [18] and might not be wholly reflected in the presented data. Another possible reason could be that in German EMS, potentially reversible causes of cardiac arrest in trauma are not addressed urgently or recognised early. Buschmann et al. showed in data of trauma-related death in the EMS in Berlin, that a reasonable proportion of patients died due to reversible causes, e.g. tension pneumothorax [19]. In contrast, new invasive techniques were implemented in the pre-hospital and early in-hospital treatment of severely injured patients. For example, Schimrigk published survival data on patients who suffered OHCA secondary to penetrating trauma and underwent resuscitative thoracotomy [20]. Implementing the REBOA technique to treat life-threatening bleeding by obstructing the aorta until arrival in hospital and start of emergency surgery has shown a benefit in terms of short-term survival [10, 11]. This study showed that patients admitted to hospital with ongoing CPR had an inferior outcome. The survival rate in that group was around 1.5%. That underlines the importance of dispatching an emergency physician with experience in invasive techniques targeting special reversible causes towards ROSC on scene.

In terms of hospital outcomes, in-hospital treatment of trauma patients is not captured in detail in the German Resuscitation Registry. Thus, the combination of data from GRR and TR-DGU was used to elucidate hospital treatment and outcome and to analyse cardiac arrest and trauma surgery items. In the present study, most patients who survived were younger than 60 and were mainly male. Patients suffering OHCA due to trauma were ten years younger than patients suffering OHCA due to other causes [2]. ROSC occurred in only 28.7% of all patients found in cardiac arrest due to trauma. Compared to Gräsner et al., survival after traumatic cardiac arrest has not improved in Germany since 2011 and is significantly lower than the average survival rate from other causes of cardiac arrest [3].

CPR during the ER treatment and age above 80 years were high-risk factors for in-hospital mortality after traumatic cardiac arrest. Head injury, initial OHCA, blood transfusion, shock on admission, and age between 70 and 79 were classified as relevant risk factors. These results are similar to the previously reported risk-adjustment scores of the TR-DGU [21, 22]. Only the initial rhythm significantly influenced mortality when analysing the GRR variables based on the Utstein-dataset for cardiac arrest. The other investigated variables showed no relevant influence in that particular subgroup. Similar results were presented in a study by Beck and colleagues, which analysed the association between Utstein factors and survival in traumatic OHCA in the Victorian Ambulance Cardiac Arrest Registry (VACAR) [23].

## Limitations

This was a combined analysis using data from two independent registries. Direct matching of cases was impossible because individual cases could not be identified in both registries. The registries had different stakeholders, voluntary participation of pre-hospital services on one side, and a network of trauma hospitals on the other. However, each registry represented a representative selection of cases. Some variables (including outcome) had missing data to various degrees. An analysis of missingness was performed. As for most data from GRR and TR-DGU data availability was > 90% or even > 95% of the cases. For the few variables with more than 10% missing the mortality rates showed no clinically relevant difference. The largest number of missing values were found in GRR for the hospital discharge rates. This high number was because not all hospitals provide survival data to the participating EMS (and thus to the GRR). Those hospitals that provide survival data provided this information for most cases. Therefore, the absence of entire hospitals was not expected to result in systematic attrition.

Due to the small number of surviving patients, and a high proportion of missing outcome information, as well as the various causes of traumatic cardiac arrest (e.g., tension pneumothorax, massive bleeding, brain injury), the conclusions were subject to some uncertainty and not allowing for firm conclusions. Due to major differences in EMS and hospital services, the present results are valid in Germany and not necessarily transposable to other countries or parts of the world.

## Conclusion

A traumatic cardiac arrest is an infrequent event with a poorer prognosis than out-of-hospital cardiac arrest due to a cardiac cause. The mortality has remained unchanged over the last decades in Germany, but 5% of all patients still survived that event. More effort is necessary to identify the reversible cause of cardiac arrest and provide targeted trauma resuscitation on scene.

## Supplementary Information

**Additional file 1:** **Table S1.** Survivor versus non-survivor in all patients with traumatic CA and CPR started (*n*=949; source: GRR; 100 patients with missing outcome status excluded).

**Additional file 2:** **Table S2.** Survivor versus non-survivor in all patients with traumatic CA, CPR started and admission to hospital with ROSC (*n*=183; source: GRR, 57 cases with missing outcome status excluded).

**Additional file 3:** **Table S3.** Final model of the multivariate logistic regression analysis to predict mortality in patients admitted to hospital with ROSC (*n*=152; source: GRR).

## Data Availability

The data that support the findings of this study are available from the GRR and the TR-DGU through an application to the registry based on the regulation of each registry.

## Abbreviations

ACSCOT: American College of Surgeons
AIS: Abbreviated Injury Scale
ASA: American Society of Anesthesiologists Physical Status Classification System
AUC: Akademie der Unfallchirurgie GmbH
BP: Blood pressure
CA: Cardiac arrest
CI: Confidence interval
CPC: Cerebral performance categories
CPR: Cardiopulmonary resuscitation
CRASS: CaRdiac-Arrest-Survival-Score
DGAI: Deutsche Gesellschaft für Anästhesiologie und Intensivmedizin, (German Society of Anaesthesiology and Intensive Care Medicine)
DGU: Deutsche Gesellschaft für Unfallchirurgie (German Society for Trauma Surgery)
ECG: Electrocardiogram
ER: Emergency room
EMS: Emergency medical services
ERC: European Resuscitation Council
EuReCa: European Registry of Cardiac arrest
FR: First responder
GOS: Glasgow Outcome Score
GRR: German Resuscitation Registry
HR: Heart rate
ICU: Intensive care unit
ISS: Injury Severity Score
LOS: Length of stay
NAEMSP: National Association of EMS physicians
NIS: Committee on Emergency Medicine, Intensive Care and Trauma Management
OHCA: Out-of-hospital cardiac arrest
OR: Odds ratio
PEA: Pulseless electrical activity
PVS: Persistent vegetative state
RACA: ROSC-after-cardiac-arrest-score
REBOA: Resuscitative endovascular balloon occlusion of the aorta
ROSC: Return of spontaneous circulation
SD: Standard deviation
TARN: Trauma Audit and Research Network
TR-DGU: Trauma Registry of the German Society for Trauma Surgery
VACAR: Victorian Ambulance Cardiac Arrest Registry
VF: Ventricular fibrillation
