# Supplementary material for: Survival after traumatic cardiac arrest is possible—a comparison of German patient-registries
Source: BMC Emerg Med. 2022 Sep 10;22:158. doi: 10.1186/s12873-022-00714-5 (PMC9463728; doi:10.1186/s12873-022-00714-5)
Supplement: Supplementary file 1 — Additional file 1: Table S1. Survivor versus non-survivor in all patients with traumatic CA and CPR started (n=949; source: GRR; 100 patients with missing outcome status excluded). [file 12873_2022_714_MOESM1_ESM.docx]

**Table S1**

Survivor versus non-survivor in all patients with traumatic CA and CPR started (n=949; source: GRR; 100 patients with missing outcome status excluded)

|  | Survivor  n=47 | Non-survivor  n=902 | p-value |
| --- | --- | --- | --- |
| Age in years^a^ | 49 [32-72] | 55 [35-73] | 0.408 |
| Male sex | 78.7% | 75.5% | 0.381 |
| Scene of cardiac arrest   - home - nursing home - workplace - street - public place - medical institution^#^ - public event - other | 38.3%  0%  6.4%  40.4%  10.6%  4.3%  0%  0% | 19.1%  2.1%  4.6%  56.7%  10.3%  2.0%  0.2%  5.0% | 0.034 |
| ECG   - VF - PEA - asystole | 15.6%  51.1%  33.3% | 3.5%  27.5%  69.0% | p<0.001 |
| CA witnessed   - by lay persons - by EMS - Not witnessed | 51.1%  21.3%  27.7% | 41.7%  11.5%  46.8% | 0.018 |
| bystander CPR | 31.9% | 30.7% | 0.861 |
| ROSC | 100.0% | 20.6% | --- |
| Time from call to EMS arrival in min^b^ | 8.8 (5.1) | 8.8 (4.9) | 0.971 |
| Time on scene in min^b^ | 41.5 (21.3) | 36.5 (13.7) | 0.079 |
| Time from accident to hospital admission in min^b^ | 64.0 (21.3) | 58.4 (18.6) | 0.076 |
| Time from CPR started to 1. ROSC in min (n=155)^b^ | 13.4 (11.4) | 21.2 (19.8) | 0.005 |

^#^ includes doctors' offices and smaller rehabilitation clinics and affiliated hospitals that do not provide their own resuscitation team but alert the EMS in such a case

Continuous measurements are presented as ^a^median [quartiles] or ^b^mean (SD)

CA, cardiac arrest; CPR, cardiopulmonary resuscitation; ECG, electrocardiogram; EMS, emergency medical service; GRR, German Resuscitation Registry; PEA, pulseless electrical activity; ROSC, return of spontaneous circulation; VF, ventricular fibrillation
