# Supplementary material for: Survival after traumatic cardiac arrest is possible—a comparison of German patient-registries
Source: BMC Emerg Med. 2022 Sep 10;22:158. doi: 10.1186/s12873-022-00714-5 (PMC9463728; doi:10.1186/s12873-022-00714-5)
Supplement: Supplementary file 2 — Additional file 2: Table S2. Survivor versus non-survivor in all patients with traumatic CA, CPR started and admission to hospital with ROSC (n=183; source: GRR, 57 cases with missing outcome status excluded). [file 12873_2022_714_MOESM2_ESM.docx]

**Table S2**

Survivor versus non-survivor in all patients with traumatic CA, CPR started and admission to hospital with ROSC (n=183; source: GRR, 57 cases with missing outcome status excluded)

|  | Survivor  n=45 | Non-survivor  n=138 | p-value |
| --- | --- | --- | --- |
| Age in years^a^ | 50 [32-73] | 58 [34-77] | 0.225 |
| Age   - <60 years - 60-69 years - 70-79 years - >80 years | 55.6%  13.3%  20.0%  11.1% | 51.5%  8.8%  19.9%  19.9% | 0.520 |
| Male sex | 77.8% | 70.3% | 0.331 |
| Scene of cardiac arrest   - home - nursing home - workplace - street - public place - medical institution^#^ - public event - other | 37.8%  0%  6.7%  42.2%  8.9%  4.4%  0%  0% | 27.5%  2.9%  8.0%  44.9%  10.1%  2.2%  0.7%  3.6% | 0.728 |
| ECG   - VF - PEA - asystole | 16.3%  51.2%  32.6% | 7.4%  44.1%  48.5% | 0.084 |
| CA witnessed   - by lay persons - by EMS - Not witnessed | 51.1%  20.0%  28.9% | 43.5%  13.8%  42.8% | 0.228 |
| Found in CA (by FR or EMS)   - Yes - No | 80.0%  20.0% | 86.2%  13.8% | 0.313 |
| bystander CPR | 33.3% | 24.6% | 0.253 |
| Shock on admission   - Yes - No - Missing (n) | 15.8%  84.2%  7 | 30.3%  69.7%  19 | 0.080 |
| Time from call to EMS arrival in min^b^ | 8.9 (5.1) | 8.9 (4.3) | 0.769 |
| Time on scene in min^b^ | 41.2 (17.1) | 38.7 (13.0) | 0.628 |
| Time from accident to hospital admission in min^b^ | 63.6 (21.4) | 62.3 (17.9) | 0.725 |
| Time from CPR started to 1. ROSC in min (n=126)^b^ | 13.4 (11.4) | 20.2 (19.7) | 0.010 |

^#^ includes doctors' offices and smaller rehabilitation clinics and affiliated hospitals that do not provide their own resuscitation team but alert the EMS in such a case

Continuous measurements are presented as ^a^median [quartiles] or ^b^mean (SD)

CA, cardiac arrest; CPR, cardiopulmonary resuscitation; EMS, emergency medical service; FR, first responder; GRR, German Resuscitation Registry; PEA, pulseless electrical activity; ROSC, return of spontaneous circulation; VF, ventricular fibrillation
