# Supplementary material for: Survival after traumatic cardiac arrest is possible—a comparison of German patient-registries
Source: BMC Emerg Med. 2022 Sep 10;22:158. doi: 10.1186/s12873-022-00714-5 (PMC9463728; doi:10.1186/s12873-022-00714-5)
Supplement: Supplementary file 3 — Additional file 3: Table S3. Final model of the multivariate logistic regression analysis to predict mortality in patients admitted to hospital with ROSC (n=152; source: GRR). [file 12873_2022_714_MOESM3_ESM.docx]

**Table S3**

Final model of the multivariate logistic regression analysis to predict mortality in patients admitted to hospital with ROSC (n=152; source: GRR)

| **Variable** | **Unit** | **OR** | **95% CI** |
| --- | --- | --- | --- |
| Age  (reference 0-59) | 60-69  70-79  80+ | 0.99  0.96  2.31 | 0.26-3.78  0.34-2.68  0.65-8.25 |
| Sex | male | 0.74 | 0.27-1.98 |
| Bystander CPR | yes | 0.75 | 0.30-1.87 |
| ECG | VF | 0.27 | 0.08-0.99 |
| Found in CA | yes | 1.76 | 0.60-5.15 |
| shock ad admission | yes | 2.48 | 0.90-6.82 |
| Location | home | 0.68 | 0.27-1.69 |
| Constant |  | 2.54 |  |

CA, cardiac arrest; CPR, cardiopulmonary resuscitation; ECG, electrocardiogram; GRR, German Resuscitation Registry; ROSC, return of spontaneous circulation
